# Integrative taxonomy of a new cocculinid limpet dominating the Aurora Vent Field in the central Arctic ocean

**DOI:** 10.1098/rsos.220885

**Published:** 2022-10-12

**Authors:** Chong Chen, Ana Hilário, Clara F. Rodrigues, Eva Ramirez-Llodra

**Affiliations:** ^1^ X-STAR, Japan Agency for Marine-Earth Science and Technology (JAMSTEC), 2-15 Natsushima-cho, Yokosuka, Kanagawa 237-0061, Japan; ^2^ Centre for Environmental and Marine Studies & Department of Biology, University of Aveiro, 3810-193 Aveiro, Portugal; ^3^ Norwegian Institute for Water Research, Oslo, Norway; ^4^ REV Ocean, Lysaker, Norway

**Keywords:** *Cocculina*, cocculinidae, Gastropoda, mollusca, gakkel ridge

## Abstract

Deep-sea hydrothermal vents host lush chemosynthetic communities, dominated by endemic fauna that cannot live in other ecosystems. Despite over 500 active vents found worldwide, the Arctic has remained a little-studied piece of vent biogeography. Though located as early as 2001, the faunal communities of the Aurora Vent Field on the ultra-slow spreading Gakkel Ridge remained unsampled until recently, owing to difficulties with sampling on complex topography below permanent ice. Here, we report an unusual cocculinid limpet abundant on inactive chimneys in Aurora (3883–3884 m depth), describing it as *Cocculina aurora* n. sp. using an integrative approach combining traditional dissection, electron microscopy, molecular phylogeny, and three-dimensional anatomical reconstruction. Gross anatomy of the new species was typical for *Cocculina*, but it has a unique radula with broad, multi-cuspid rachidian where the outermost lateral is reduced compared to typical cocculinids. A phylogenetic reconstruction using the mitochondrial COI gene also confirmed its placement in *Cocculina*. Only the second cocculinid found at vents following the description of the Antarctic *Cocculina enigmadonta*, this is currently the sole cocculinid restricted to vents. Our discovery adds to the evidence that Arctic vents host animal communities closely associated with wood falls and distinct from other parts of the world.

## 1. Introduction

Since hydrothermal vents were first discovered on the Galápagos Rift in 1977 [1], ecosystems powered by bacterial chemosynthesis, where energy is derived from reducing compounds such as methane and hydrogen sulfide, have become a focus of biological research [2]. Chemosynthesis-based ecosystems such as hydrothermal vents, hydrocarbon seeps and organic falls like wood- and whale falls can host very high levels of biomass owing to local primary production. Most species in such ecosystems have adapted so much to their specific habitats that they are endemics that do not occur anywhere else [3]. Chemosynthetic systems in different parts of the world exhibit distinctive species composition, and accumulating data from hydrothermal vents revealed the existence of distinctive vent biogeographic provinces [4,5].

Despite more than 500 active vents having been located around the globe with over 600 animal species found to inhabit them [6,7], a number of understudied geographical ‘missing pieces’ preclude us from understanding the global hydrothermal vent biogeography [8]. One of these regions is the Central Arctic Ocean, which was hypothesized to host completely different vent animals from other regions owing to the shallow sill generated by the Iceland hotspot preventing any deep connection between it and other major oceans [8]. The first explorations of Arctic vents took place on the Mohn's Ridge after venting was located there in 2005, in the 550–750 m deep Jan Mayen vent fields just north of 71° N. Then, the first deep (2400 m) vents were located at Loki's Castle at 73° N in 2008 [9,10]. Unlike the Mid-Atlantic Ridge vents south of Iceland, where dense aggregations of alvinocaridid shrimps and bathymodioline mussels dominate the communities, these animals were completely lacking on the Mohn's Ridge. The Jan Mayen vents lack any significant vent-endemic fauna and are dominated by the grazing rissoid snail *Pseudosetia griegi* (Friele, 1879) [11], originally described from a piece of sunken wood [10]. Though shallow water vents typically lack endemic fauna, they would usually be present in those over 500 m deep [6,7]. Loki's Castle is dominated by the cosmopolitan symbiotic tubeworm *Sclerolinum contortum* Smirnov, 2000 and gastropods, but otherwise also lacked typical vent fauna of the Mid-Atlantic Ridge [9,12]. These observations appear to support the isolation of Arctic vents, and that they are dominated by locally adapted fauna.

Nevertheless, the picture is incomplete with a lack of data from the ultra-slow-spreading Gakkel Ridge in the Central Arctic Ocean. Although evidence of hydrothermal venting in the southern Gakkel Ridge was obtained from hydrothermal plume signals and the collection of pieces of sulfide chimney as early as 2001 [13], the presence of black smokers was only confirmed in 2014, during the R/V *Polarstern* cruise PS86, on an active site named the Aurora Vent Field (AVF; 82°53.83′ N, 6°15.33′ W, 3880 m depth) on the Aurora mound [14]. This is the northernmost confirmed vent site to date [7], covered by a permanent layer of Arctic sea ice. The images obtained during the PS86 cruise and additional images obtained during the HACON19 cruise on board R/V *Kronprins Haakon* in 2019 using the towed Ocean Floor Bathymetry and Observation System showed a high density of white limpet-like and snail-like organisms, and a lower density of melitid amphipods [14,15]. Attempts to sample the AVF, however, have been hindered owing to the challenges associated with sampling deep-sea hydrothermal vents under permanent ice cover. Finally, in 2021, a full survey and comprehensive sampling of the AVF was conducted with REV Ocean's remotely operated vehicle (ROV) *Aurora* during the HACON21 cruise on-board R/V *Kronprins Haakon* [16]. The biological samples revealed the limpet-like organisms previously seen on cameras to be a cocculinid limpet.

Cocculinidae is a family of limpet-formed gastropods in the superfamily Cocculinoidea (subclass Neomphaliones, order Cocculinida) [17]. The majority of the species are associated with organic falls, most commonly with sunken wood, although how they obtain nutrition from these substrates remains debated [18–20]. There are currently six described genera in the family: *Cocculina, Coccopigya, Coccocracter, Paracocculina,* and *Macleaniella*, distinguishable by shell sculpture and shape, as well as the condition of the copulatory organ and epipodial tentacles [21–24]. Two genera, *Fedikovella* and *Teuthirostria*, were traditionally considered to be cocculinids, but were recently transferred to a new family, Teuthirostriidae, based on anatomical and protoconch characters [23]. The nominal genus of Cocculinidae is *Cocculina*, containing about 30 named species around the globe, primarily inhabiting sunken wood. Three species have been collected from whale and dolphin carcasses [25], including *Cocculina enigmadonta* Chen & Linse, 2020 [26] from the Southern Ocean, which is also found on a hydrothermal vent at Kemp Caldera on the South Sandwich Arc, making it the only known vent cocculinid until now [26]. The phylogenetic position of *C. enigmadonta,* nested within sunken wood species, has been suggested as an example of a species using organic falls as ‘stepping-stones’ to adapt to a more ‘extreme’ hot vent habitat [26,27].

Here, we characterize the AVF cocculinid using an integrative approach combining dissection, electron microscopy, three-dimensional anatomical reconstruction, and molecular phylogeny, in order to provide a formal description and to gain insights on its evolutionary history and ecology.

## 2. Materials and methods

### 2.1. Sample collection

**Figure 1.**
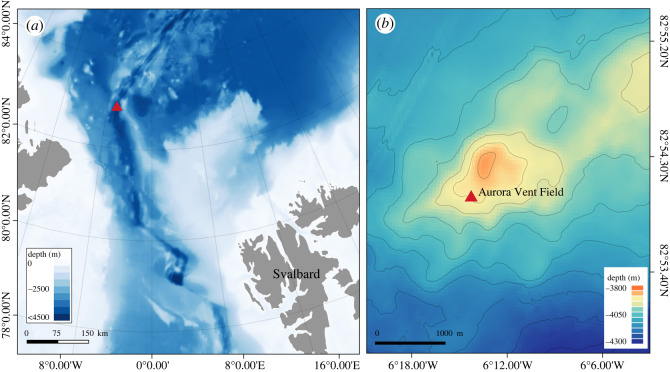
Map of the Aurora Vent Field (AVF), indicated by a red triangle. (*a*) Overview map showing general location of the AVF. (*b*) Close-up bathymetric map of the Aurora mound showing the AVF located southwest of the summit. Map generated by Sofia Ramalho, University of Aveiro.

**Figure 2.**
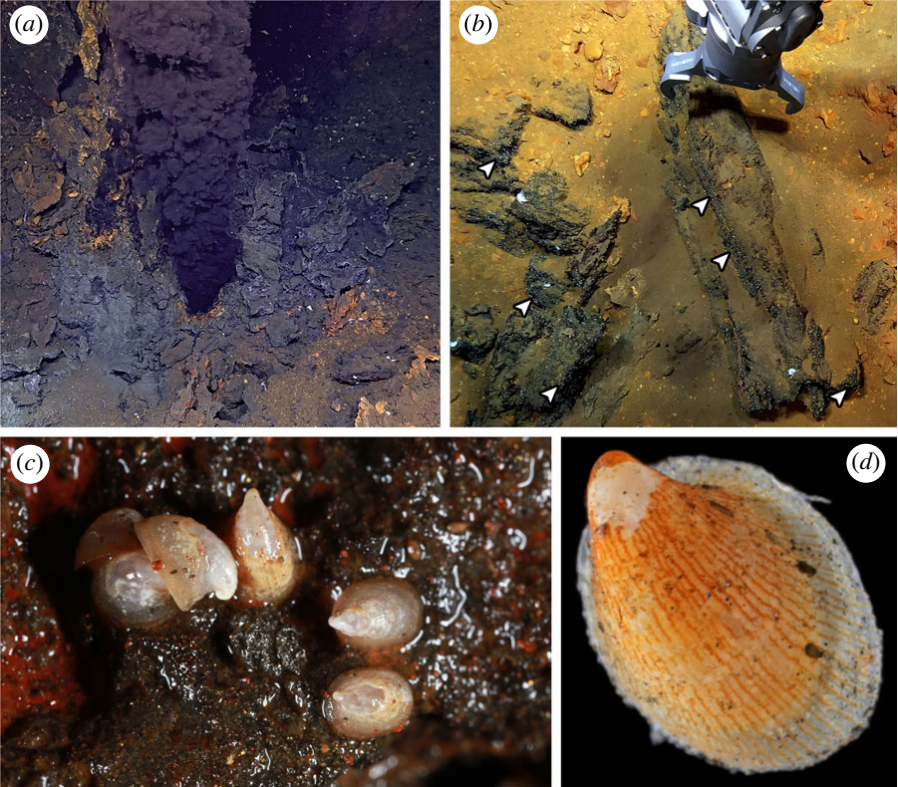
The Aurora Vent Field (AVF) and the cocculinid limpet. (*a*) Enceladus black smoker, AVF. (*b*) ROV *Aurora* collecting a piece of sulfide chimney from the AVF; white dots indicated by arrows are cocculinid limpets while the larger white animals are amphipods. (*c*) Live ccculinid limpets on chimney surface after recovery on-board. (*d*) A live cocculinid limpet viewed under a dissecting microscope.

Cocculinid limpets were collected from inactive sulfide rocks at the base of the Hans Tore Vent (82°53.8307′ N, 6°15.3251′ W, 3883 m deep) and Ganymede (82°53.8267′ N, −6°15.3608′ W, 3884 m deep) black smokers of the AVF, Gakkel Ridge (figures 1 and 2*a,b*) by ROV *Aurora,* during the HACON21 cruise on-board R/V *Kronprins Haakon* in October 2021 [16]. Upon recovery of dead-chimney pieces on-board, cocculinid limpets attached to the sulfide rocks (figure 2*c,d*) were removed and either fixed in 4% paraformaldehyde for 48 h and then transferred to 70% ethanol, or preserved directly in 96% ethanol. Specimens used herein were deposited in the Natural History Museum, London (NHMUK), the Biological Research Collection (Marine Invertebrates) of the Department of Biology of the University of Aveiro (CoBI-DBUA), and the Mollusca collection of the National Museum of Nature and Science, Tsukuba (NSMT-Mo), Japan.

### 2.2. Gross morphology

Limpets were observed under a stereo dissecting microscope (Olympus SZX9), with photographs of the specimens taken using a digital single-lens reflex camera (Olympus E-M5 Mark III) attached to the trinocular. Shell length (SL), shell width (SW) and shell height (SH) measurements were taken using digital callipers and rounded up to the nearest one decimal point. For observation of external anatomy, soft parts were gently removed from the shell, cleaned using fine brushes and dissected using fine forceps and tungsten needles.

### 2.3. Scanning electron microscopy

For scanning electron microscopy (SEM) observations of the periostracum, shells were cleaned using a fine brush and directly mounted by carbon tape on aluminium stubs. To observe protoconch and teleoconch sculpture, shells were placed in diluted (10%) household bleach for 30 s and cleaned by a fine brush to remove debris and also the periostracum. The cleaned shells were washed for a few times in MilliQ water and twice in 99% ethanol, before being mounted on aluminium stubs. For radula observations, the radula ribbon was dissected from the limpets using tungsten needles and then cleaned in 10% household bleach to dissolve any remaining soft tissue. The cleaned radulae were then washed and mounted in the same manner as shells. All SEM stubs prepared were observed uncoated at 15 kV using a Hitachi TM-3000 tabletop SEM.

### 2.4. Three-dimensional anatomical reconstruction

A formaldehyde-fixed and 70% ethanol-preserved limpet was used for three-dimensional anatomical reconstruction using X-ray micro-computed tomography (μ-CT). The soft parts were removed from the shell using tungsten needles, and then rehydrated through a graded ethanol-MilliQ water series (70%, 50%, 30%, 10%, 0% ethanol) for 1 h each and then left to sit in fresh MilliQ water overnight. The rehydrated limpet was stained for 48 h in 1% iodine solution prior to the CT scan. The stained specimen was washed twice in MilliQ water and placed inside an X-ray transparent polystyrene container filled with MilliQ water, as per published methods [28]. The specimen was scanned wet using a ScanXmate-D160TSS105 (Comscantecno, Japan) commercial μ-CT at 80 kV/51 µA with the resolution of 3.150 µm per pixel, at 992 pixels by 992 pixels per slice. The images obtained were contrast-enhanced and cropped in Adobe Photoshop CC, then imported to the specialist software Amira v2019.1 (Thermo Fisher Scientific). The organs of interest were highlighted manually in Amira, and their three-dimensional surfaces were rendered as per published methods [29]. The rendered surfaces were complexity-reduced and smoothed to generate the final tomographic model for display.

### 2.5. DNA extraction, amplification and sequencing

Genomic DNA was extracted from three limpet specimens using the ISOLATE II Genomic DNA Kit (Bioline), following manufacturer's protocols. The universal primer pair LCO1490 and HCO2198 [30] was used to amplify the barcoding fragment of the mitochondrial cytochrome *c* oxidase subunit I (COI) gene. Polymerase chain reaction (PCR) was carried out using the Supreme NZYTaq II DNA polymerase (NZYTech) in a total volume of 25 µl: 12.5 µl buffer, 1 µl template DNA, 1 µl of each primer (10 mM) and topped up with 9.5 µl of water. The PCR programme was 94°C for 4 min followed by 35 cycles (94°C for 1 min, 50°C for 1 min and 72°C for 1 min), ending with 72°C for 7 min. Amplification was confirmed using 1% agarose gel electrophoresis and the PCR product was purified using the ISOLATE II PCR and Gel Kit (Bioline) following the manufacturer's standard protocols. Sequencing of both forward and reverse strands was done in GATC services at Eurofins Genomics (Germany).

### 2.6. Phylogenetic reconstruction

Forward and reverse COI sequences of the AVF cocculinids obtained were aligned and manually corrected. The consensus sequences were aligned with GenBank sequences of gastropods in the order Cocculinida, plus additional sequences from its sister-clade Neomphalida, as well as morphologically convergent pseudococculinid limpets [18,21,31] in the vetigastropod order Lepetellida, using Geneious R11 (https://www.geneious.com/). The caenogastropod *Neptunea antiqua* (Linnaeus, 1758) was used as an outgroup to root the phylogeny following a previous study [26]. Phylogenetic reconstruction was carried out using Bayesian inference by the software MrBayes v3.2.6 [32] using a 621 bp alignment of the COI gene, with the HKY + Gamma model as selected by the Bayesian information criterion in PartitionFinder 2 [33]. In the reconstruction, metropolis-coupled Monte Carlo Markov chains were run for two million generations, topologies were sampled every 100 generations and the first 25% were discarded as burn-in. Tracer v1.7 [34] was used to confirm that split frequencies were below 0.01. New sequences generated herein have been deposited in NCBI GenBank under the accession numbers: ON873626–ON873628.

## 3. Results

### 3.1. Systematics

Order Cocculinida Thiele, 1909

Superfamily Cocculinoidea Dall, 1882

Family Cocculinidae Dall, 1882

Genus *Cocculina* Dall, 1882

**Figure 3.**
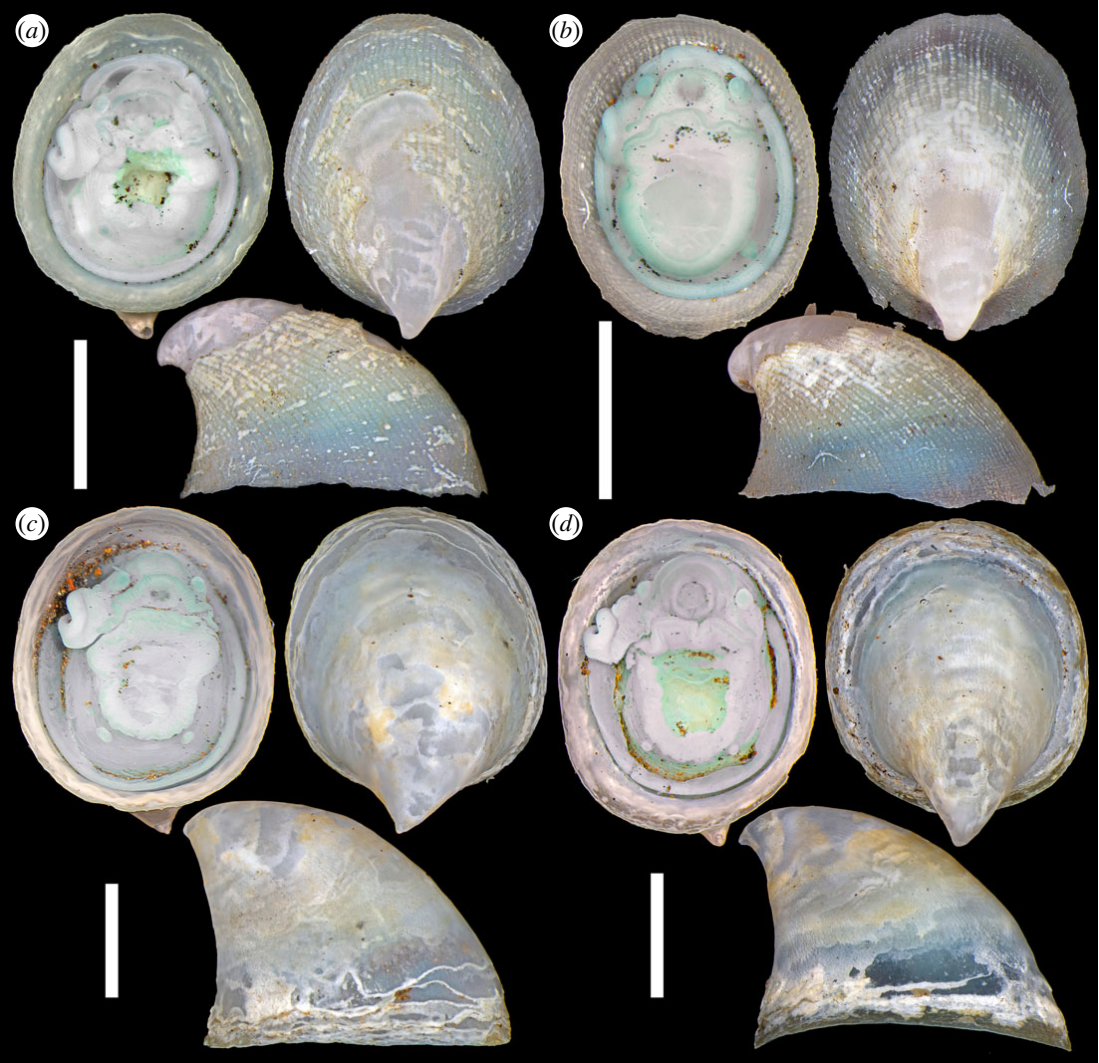
*Cocculina aurora* n. sp., representative type specimens in dorsal, ventral and lateral views. Specimens have been cleaned by a soft brush prior to photography. (*a*) Holotype (NHMUK 20220343). (*b*) Paratype 1 (CoBI-DBUA2532.01.v01). (*c*) Paratype 2 (NHMUK 20220344). (*d*) Paratype 3 (NSMT-Mo 79370). Scale bars, 2 mm.

**Figure 4.**
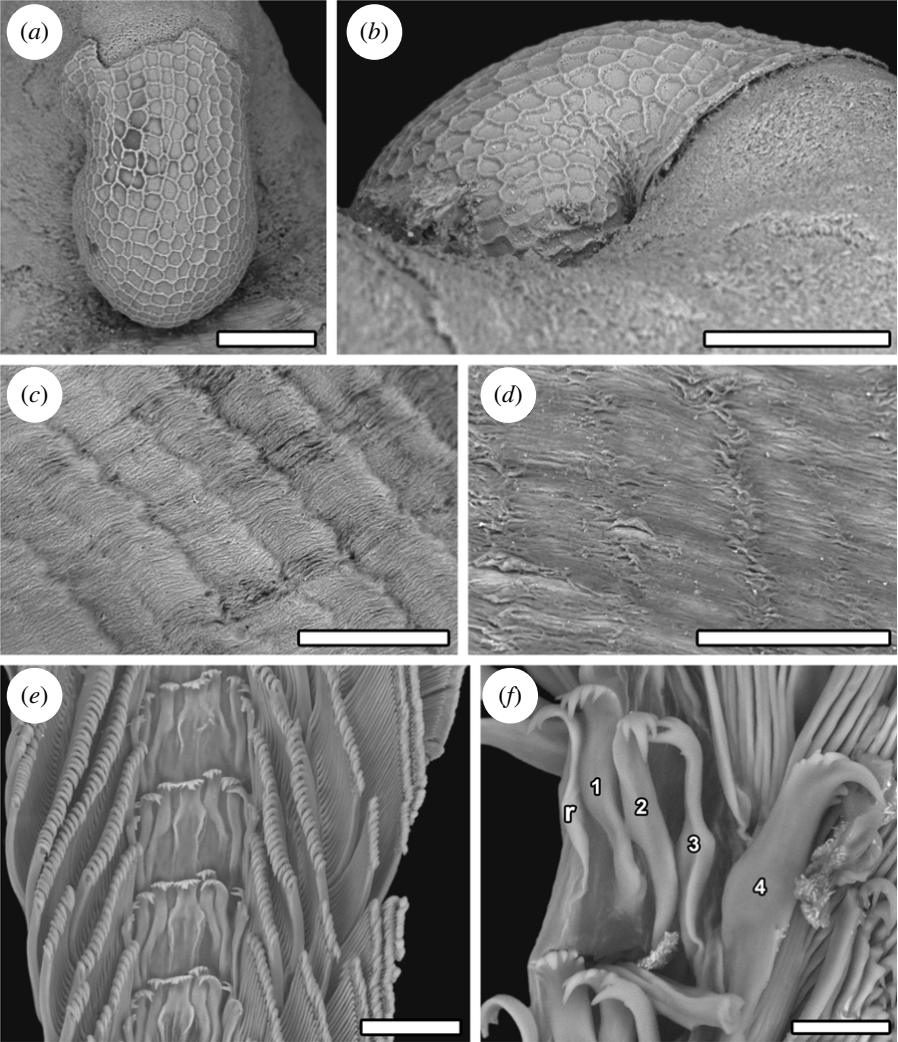
*Cocculina aurora* n. sp. (paratype 6, NSMT-Mo 79371), scanning electron micrographs. (*a*–*b*) Protoconch. (*c*) Details of the teleoconch shell sculpture after removing the periostracum. (*d*) Details of the periostracum. (*e–f*) Radula, r = rachidian, numbers represent the number of lateral teeth. Scale bars, (*a,b,d*), 100 µm; (*c*), 150 µm; (*e*), 50 µm; (*f*), 20 µm.

**Figure 5.**
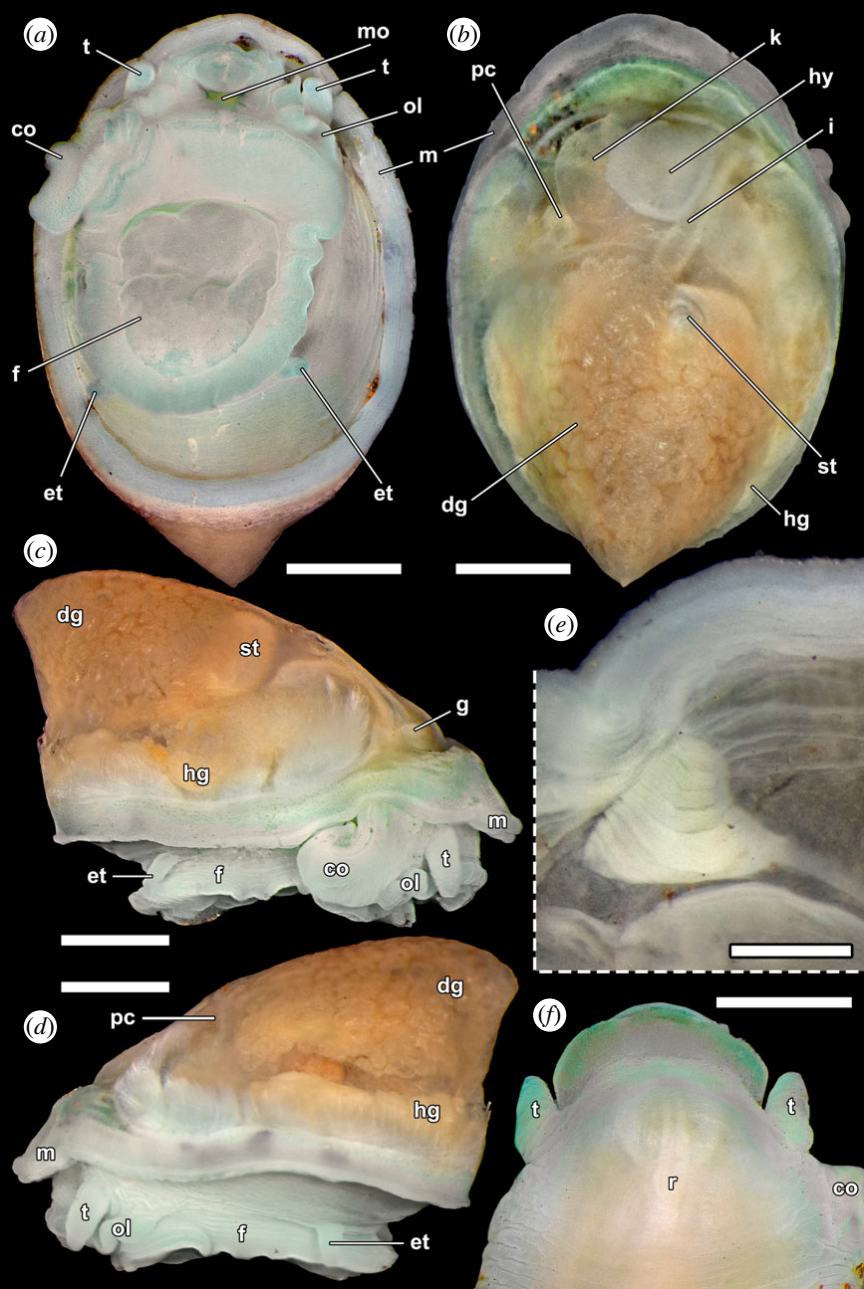
*Cocculina aurora* n. sp. (paratype 7, NSMT-Mo 79372), external anatomy after shell removed. (*a*) Ventral view. (*b*) Dorsal view. (*c,d*) Lateral views. (*e*) Pseudoplicate gill on the right mantle roof viewed ventrally after cutting away tissue along the dotted line. (*f*) Dorsal view of the head after removal of the mantle. Abbreviations: co = copulatory organ, dg = digestive gland, et = epipodial tentacle, f = foot, g = pseudoplicate gill, gd = gonoduct, hg = hermaphroditic gland, hy = hypobranchial gland, k = kidney, m = mantle edge, mo = mouth, ol = oral lappet, pc = pericardium, r = radula, st = stomach, t = cephalic tentacle. Scale bars, (*a–d*), 1 mm; (*e*), 0.1 mm; (*f*), 0.5 mm.

**Figure 6.**
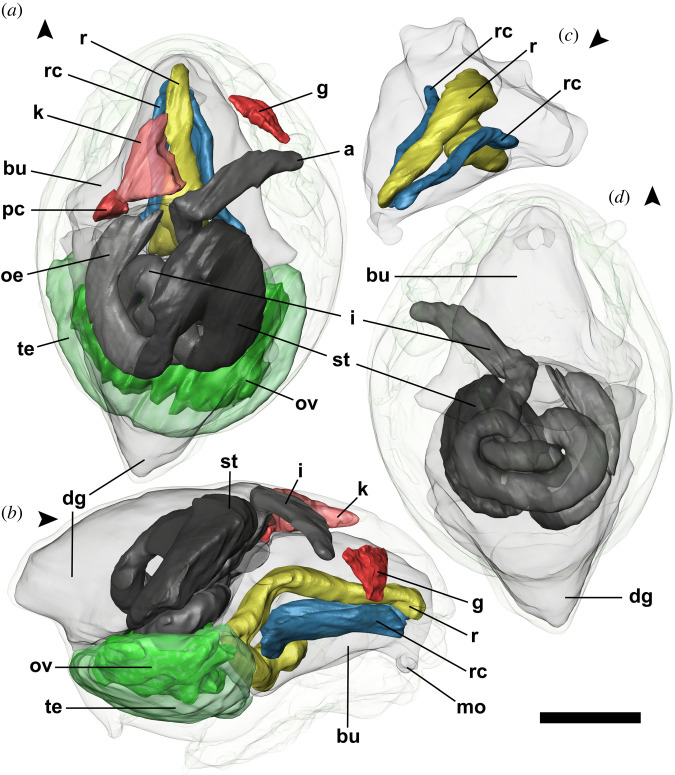
*Cocculina aurora* n. sp. (paratype 8, NSMT-Mo 79373), three-dimensional reconstruction of the gross internal anatomy. Anterior direction of the animal is indicated by an arrowhead for each part of the figure. (*a*) Dorsal view showing all organs reconstructed. (*b*) Lateral view showing all organs reconstructed. (*c*) Radula apparatus shown inside the buccal mass. (*d*) Ventral view showing the alimentary tract. Abbreviations: a = anus, bu = buccal mass, dg = digestive gland, g = pseudoplicate gill, i = intestine, k = kidney, mo = mouth, ov = ovary part of the hermaphroditic gland, oe = oesophagus, pc = pericardium, r = radula, rc = radula cartilage, st = stomach, te = testis part of the hermaphroditic gland. Scale bar, 1 mm, applies to the entire figure.

***Cocculina aurora* n. sp.**

(Figures 2*c,d*, 3–6)

*ZooBank registration LSID*: urn:lsid:zoobank.org:act:779A806E-6BB1-4236-8EA9-C3B274B6401F

*Diagnosis.* A medium-sized *Cocculina* (up to 6.5 mm in SL) with tall, cap-like shell whose apex extends to or beyond the posterior aperture edge. Protoconch with reticulate sculpture, teleoconch with faintly raised radial ribs. Copulatory organ on right side of the oral lappet. Radula with multi-cuspid and broad central tooth, outermost lateral teeth only slightly larger than other laterals, over 40 marginal teeth on each side.

*Type locality*. On inactive sulfide deposits and dead chimneys, 3883–3884 m deep on the AVF (82°53.8′ N, 6°15.3′ W), Gakkel Ridge, Arctic Ocean.

*Type materials*. Holotype (NHMUK 20220343; figure 3*a*), SL 4.5 mm, SW 3.3 mm, live collected, fixed in 4% formaldehyde and stored in 70% ethanol, Hans Tore Vent chimney (82°53.8307′ N, 6°15.3251′ W, 3883 m deep), AVF, Gakkel Ridge, ROV *Aurora* dive no. 12, 2021/x/08, R/V *Kronprins Haakon* cruise HACON21. Paratype 1 (CoBI-DBUA2532.01.v01; figure 3*b*), SL 3.6 mm, SW 2.8 mm, same preservation and data as holotype. Paratype 2 (NHMUK 20220344; figure 3*c*), SL 5.8 mm, SW 4.5 mm, same preservation and data as holotype. Paratype 3 (NSMT-Mo 79370; figure 3*d*), SL 5.3 mm, SW 3.9 mm, same preservation and data as holotype. Paratype 4 (CoBI-DBUA2532.01.v02), SL 6.5 mm, SW 4.2 mm, same preservation and data as holotype. Paratype 5 (NHMUK 20220345), SL 4.6 mm, SW 3.3 mm, same preservation and data as holotype. Paratype 6 (NSMT-Mo 79371; figure 4), SL 3.8 mm, SW 3.0 mm, same preservation and data as holotype, dissected for SEM. Paratype 7 (NSMT-Mo 79372; figure 5), SL 4.7 mm, SW 3.5 mm, same preservation and data as holotype, soft parts dissected for external anatomy. Paratype 8 (NSMT-Mo 79373; figure 6), SL 4.2 mm, SW 3.2 mm, same preservation and data as holotype, used for μ-CT scanning. Paratype 9 (CoBI-DBUA2532.01.v03-v05), a series of three specimens, preserved in 90% ethanol, same data as holotype, used for DNA sequencing. Paratype 10 (NHMUK 20220346), a series of five specimens preserved in 90% ethanol, Ganymede black smoker chimney (82°53.8267′ N, 6°15.3608′ W, 3884 m deep), AVF, Gakkel Ridge, ROV *Aurora* dive no. 16, 2021/x/10, R/V *Kronprins Haakon* cruise HACON21.

*Description*. Shell (figures 3 and 4*a–d*) medium-sized for cocculinids (maximum SL 6.5 mm), white, thin, semi-transparent. Aperture oval, slightly irregular in shape. Lateral profile low when young, becoming taller with larger size. Shell margin vary in form, presumably to fit shape of substrate (figure 3*c,d*). Anterior slope convex, posterior slope strongly concave. Apex very posterior, either at or projecting slightly beyond posterior edge of aperture. Dorsal profile drop-shaped owing to posteriorly protruding apex. Early teleoconch typically corroded, corrosion expanding to entire teleoconch surface in older individuals. Secondary thickening of apical area observed, likely to compensate for corrosion. Protoconch (figure 4*a,b*) bulbous, length approximately 300 µm, with regular, reticulate, honeycomb-like sculpture. Tip of protoconch buried in posterior coil of shell. Transition between protoconch and teleoconch marked by slightly raised lip on protoconch. Teleoconch sculptured by subtly raised radial striae between 70 and 100 µm in width (figure 4*c*), otherwise only very fine concentric growth increments present. Teleoconch covered by thin, semi-transparent layer of greenish periostracum showing finely raised growth lines especially clear on top of radial striae (figure 4*d*).

Radula (figure 4*e,f*) rhipidoglossate, formula n – 4 – r – 4 – n. Rachidian tooth broad, multi-cuspid with overhanging cusps. Central cusp strong, incompletely separated into denticles, with 4–5 weaker denticles on either side, descending in strength outwards. Shaft of rachidian broad with rectangular base, lateral ridge well-developed. All laterals robust, multi-cuspid with overhanging cusps. First lateral tooth positioned higher than rachidian, robust, with four weaker cusps to either side of central cusp. Shaft elongate, abruptly bending laterally about mid-length. Second lateral positioned at same level with rachidian, similar to first lateral except with straight shaft. Third lateral similar to second lateral except cusps being uneven, with strongest cusp positioned at outermost, secondary cusps decrease in strength inwards. Outermost lateral with straight, well-reinforced shaft, only about 1.5 times as broad as other laterals, with two secondary cusps outside of central cusp, four inside it. Marginal teeth numbering over 50 on either side, slightly diminishing in size outwards. Each marginal tooth with narrow, very elongate shaft, ending in small narrow cusp equipped with four hook-like denticles, plus one strong lateral denticle. Shafts of outer marginals exhibit mid-shaft twist.

External anatomy (figure 5). Animal whitish in colour, without trace of eyes from external observation. Cephalic tentacles simple, conical, symmetrical, projecting slightly farther than snout in preserved condition. Snout short. Radula sac visible through transparency dorsally. Ventral mouth with cuticular epithelium lining typically seen for cocculinids. Oral lappets surrounding mouth thick, laterally broadened. Copulatory organ projecting from right side of oral lappet behind right cephalic tentacle, equipped with double seminal groove. Mantle margin simple, tentacles lacking. Shell attachment muscle horseshoe-shaped, encircling posterior three-fourths of dorsal surface, divided into indistinct bundles. Single pair of simple posterior epipodial tentacles present. Pallial cavity shallow, only occupying anterior one-fourth of body length. One triangular, short, pseudoplicate gill (figure 5*e*) present on right side of pallial mantle roof, equipped with 8–10 vestigial leaflets lacking sensory bursicles. Osphradium without visible zonation, on left side of pseudoplicate gill. Posterior portion of mantle roof occupied by pericardium on left, connected to sizeable kidney near midline. Pouch-like hypobranchial gland present, positioned at right side of kidney. Dorsal aspect of visceral mass visually dominated by expansive digestive gland, with occasionally visible parts of stomach and intestine.

Operculum absent.

Internal anatomy (figure 6). Radular sac occupying approximately half of body length, at posterior abruptly turning 180° anteroventrally, forming C-shape when viewed laterally. Posterior end of radular sac weakly bifurcated with lateral projections. One lateral pair of radular cartilages present just ventral of anterior half of radular sac. Buccal mass massive, laterally expanding posteriorly, occupying half of body length. Oesophagus emerges posteriorly from buccal mass at posterior left side of body, anterodorsally running in C-shape to enter stomach. Stomach sizeable, turning posteroventrally in opposite manner as oesophagus to emerge as intestine on left side, just ventral of oesophagus. Intestine makes three tight loops before emerging anteriorly near midline, then turning abruptly towards right pallial cavity to form rectum, ending in simple anus on mantle roof just posterior of pseudoplicate gill. Digestive gland occupies nearly all of dorsal-posterior space behind and above stomach, as well as filling space among intestinal loops. Intestine and stomach contents comprising unidentified organic matter with occasional mineral particles mixed in, presumably pieces of sulfides ingested with food.

Very large hermaphroditic gland situated at posteroventral part of visceral mass, ventral of alimentary tract. Hermaphroditic gland clearly separated into parts producing egg or sperm. Ovary part of hermaphroditic gland dorsal to testis part, fully embedded in testis except dorsally. Ovary of specimen examined in μ-CT ripe with numerous yolk-rich eggs. Gonoduct with massive glands, emerging from right side of hermaphroditic gland, directed anteriorly.

*Dimensions*. Specimens investigated ranged between 1.8 mm to 6.5 mm in SL and 1.4 mm to 4.5 mm in SW. Typical size approximately 5 mm in SL and 3.5 mm in SW.

*Etymology*. A name with triple meaning alluding to Aurora, the goddess of dawn in Roman mythology, the type locality AVF and ROV *Aurora* that collected the type specimens.

*Distribution*. Only known from the type locality, where it has been collected on the surface of sulfides (dead chimneys) in close vicinity of high-temperature, vigorous venting black smokers around the Hans Tore Vent and Ganymede black smokers.

*Remarks*. The position of the copulatory organ at the right side of the oral lappet, the strongly bend posterior oesophagus at the left side of the body, a single pair of epipodial tentacles, reticulate protoconch sculpture, and simple radial teleoconch sculpture without spinose periostracal hair combined to place the present new species in genus *Cocculina* [22–24]. *Cocculina aurora* n. sp. cannot be confused with any other described cocculinids, as its radula is highly distinctive with a robust, broad, multi-cuspid rachidian combined with an outermost lateral tooth that is not dramatically more massive than the other laterals. The stark difference in the morphology of the outermost lateral teeth compared to other cocculinids is clearly visible in observations under both light microscopy and SEM.

### 3.2. Molecular phylogeny

**Figure 7.**
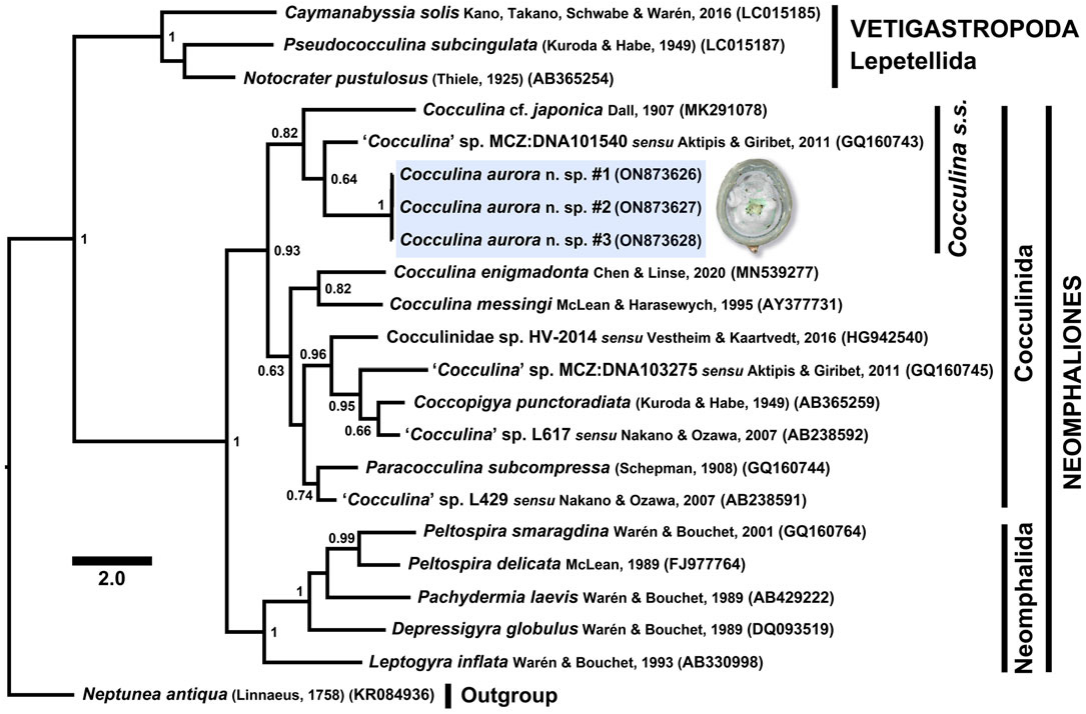
Phylogenetic reconstruction using 621 bp of the mitochondrial COI gene with Bayesian inference, showing the phylogenetic position of *Cocculina aurora* n. sp. within Cocculinidae. GenBank accession numbers are displayed in parenthesis. Node values are Bayesian posterior probabilities, only those higher than 0.6 are shown.

Phylogenetic reconstruction using the COI gene (figure 7) recovered the three sequences of *C. aurora* n. sp. within a strongly supported (Bayesian posterior probability (BPP) = 0.93) clade containing all cocculinid sequences, interpreted as a monophyletic Cocculinida. Neomphalida was recovered as a fully supported (BPP = 1) monophyletic group, sister to Cocculinida. The clade containing Cocculinida and Neomphalida was also fully supported (BPP = 1), interpreted to represent a monophyletic subclass Neomphaliones. The pseudococculinid limpets in the subclass Vetigastropoda that are morphologically convergent with cocculinids were clustered in a separate fully supported (BPP = 1) clade outside Neomphaliones.

Within Cocculinidae, *C. aurora* n. sp. was sister to an undescribed cocculinid species, ‘*Cocculina*’ sp. MCZ:DNA101540 *sensu* [35] with weak support (BPP = 0.64). This pair was in turn sister to *Cocculina* cf. *japonica* Dall, 1907, the three being united in a moderately supported (BPP = 0.82) monophyletic clade, sister to all other cocculinid sequences included. Among the other cocculinids included, *Cocculina messingi* McLean & Harasewych, 1995 and *C. enigmadonta* were recovered as sisters with moderate support (BPP = 0.82). Three undescribed ‘*Cocculina*’ species from the publications [35–37] were recovered together with *Coccopigya punctoradiata* (Kuroda & Habe, 1949) in a strongly supported (BPP = 0.96) clade; whereas another undescribed ‘*Cocculina*’ species (‘*Cocculina*’ sp. L429) from [36] was recovered sister to *Paracocculina subcompressa* (Schepman, 1908) in a moderately supported (BPP = 0.74) clade.

## 4. Discussion

### 4.1. Molecular phylogeny

Our phylogenetic reconstruction (figure 7) confirmed the placement of *C. aurora* n. sp. within Cocculinidae. The two species found clustered together with *C. aurora* n. sp. (i.e. *C. japonica* and an undescribed cocculinid) were recovered with good confidence in *Cocculina sensu stricto* in a recently published phylogeny [24], and as such we interpret the clade containing these three species as *Cocculina s.s.* This agrees with the anatomical features of *C. aurora* n. sp. and confidently places this species in genus *Cocculina*. Although the tall, cap-like shell morphology of *C. aurora* n. sp. differs substantially from typical low, flat shells of other species in this clade [24], cocculinid shell form is known to be variable across species and both the radula and the anatomy provide more reliable taxonomic characters [22,23].

*Cocculina messingi* and *C. enigmadonta* being recovered outside of *Cocculina s.s.* also agrees with the larger scale phylogeny presented in [24]. As it is at the moment unclear what morphological character separates these species from *Cocculina s.s.*, we agree with that study and leave these two species in *Cocculina sensu lato* for the time being. The five undescribed ‘*Cocculina*’ species with only sequence data available included herein were scattered within Cocculinidae, with only one species likely to actually belong to *Cocculina*, while three exhibited closer affinity with *Coccopigya* and one with *Paracocculina*. Determining the true generic affiliation of these species requires data on their morphology.

### 4.2. From wood to vent

*Cocculina aurora* n. sp. was not found to be closely related with the only other cocculinid known to inhabit hydrothermal vent ecosystems, *C. enigmadonta* from the Southern Ocean [26]. This means the invasion of hydrothermal vent ecosystems occurred at least twice in Cocculinidae, independently. Though it is of interest that the only two vent cocculinids known have been discovered from the two polar regions, one in the Arctic and the other in the Antarctic, this is probably coincidental and future explorations of vent systems elsewhere will probably bring to light additional vent cocculinids. Whereas *C. enigmadonta* is known from both hydrothermal vents and whale falls [26], *C. aurora* n. sp. is currently the sole cocculinid species known from only hydrothermal vents. However, as the deep Central Arctic Ocean has been very poorly sampled to date, there remains the possibility that it may be found on organic falls in the future.

Like *C. enigmadonta* [26], *C. aurora* n. sp. is also phylogenetically nested in a derived position within wood-fall specialist species, suggesting that it also used organic falls as evolutionary stepping-stones to adapt to living in hydrothermal vents. This adds to the evidence that such transitions have occurred many times in the contemporary vent-endemic fauna, with other examples being bathymodioline mussels and neolepetopsid true limpets [38,39]. As all other members of *Cocculina s.s.* are found either on sunken wood or cetacean carcasses [24], there is currently no evidence that hydrocarbon seeps played any role in the adaptation of vent cocculinids. However, it should be noted that the undescribed ‘Cocculinidae sp. HV-2014′ [37] was found at a brine pool in the Red Sea and probably represents a cocculinid uniquely adapted to seeps.

The gut contents of *C. aurora* n. sp. was similar to *C. enigmadonta* [26] in comprising organic matter, probably ingested bacterial mats, mixed with occasional sulfide particles; such particles were also found on the radula. This suggests *C. aurora* n. sp. feeds on bacterial mats that colonize chimney surfaces in the AVF [16], a different food source from the typical sunken wood cocculinid species which process and ingest wood pieces [22]. The highly modified radula seen in *C. aurora* n. sp. is probably linked to this ecological transition of food source. Typically, in cocculinids, the massive outermost lateral teeth dwarf all other teeth, and being equipped with multiple strong cusps, it is the major food processing tooth [18,31]. The much-reduced outermost lateral teeth in *C. aurora* n. sp. would allow the entire radula row to better contact the chimney surface, probably facilitating the sweeping of a larger surface area per rasping action [31,40]. In *C. enigmadonta*, which also exploits the same food source, a different strategy is taken where the outermost lateral teeth are still massive in size but have become very flattened and unicuspid [26], to much the same effect. Both species have convergently evolved an increased number of marginals than typical for sunken wood cocculinids, probably an additional adaptation to increase the grazed surface area. These two species exemplify that cocculinids have the capacity to flexibly modify their radula in order to adapt to novel food sources. Similar to *C. enigmadonta*, the anatomy of the alimentary tract in *C. aurora* n. sp. was not significantly modified from the typical *Cocculina* formula [22,23].

### 4.3. Significance for community structure at the Aurora Vent Field

*Cocculina aurora* n. sp. was among the most abundant benthic animals found at the AVF, alongside small-coiled snails and a lower density of melitid amphipods [16]. This faunal pattern is similar to the vent communities on the northern part of the Mohn's Ridge, where typical Mid-Atlantic Ridge vent-endemic fauna such as bathymodioline mussels and alvinocaridid shrimps are lacking [9,10], further supporting the uniqueness of Arctic vents in terms of species composition and biogeography [8]. The lack of vent-endemic fauna may have facilitated local adaptation and invasion of species like the meltid amphipod *Exitomelita sigynae* with putative symbiotic associations [41] and *Cocculina aurora* sp. nov. The vents on the Mohn's Ridge are notable in sharing taxa with wood falls, exemplified by the skeneid snail *Skenea profunda*, the rissoid snail *Pseudosetia griegi* and the meltid genus *Exitomelita* [9,42]. The finding of a vent cocculinid in the AVF extends the pattern to the Gakkel Ridge, as cocculinids are well-known from sunken wood habitats of the northern Atlantic [43]. Our discovery adds to the evidence that hydrothermal vents on the Arctic Mid-Ocean Ridges are distinctive from other regions of the world, warranting future global analyses to test the position of this ‘missing piece’ in vent biogeography.

## Data Availability

Specimens used were deposited in the Natural History Museum, London (NHMUK), the Biological Research Collection (Marine Invertebrates) of the Department of Biology of the University of Aveiro (CoBI-DBUA), or the National Museum of Nature and Science, Tsukuba (NSMT), Japan. Newly generated DNA sequences were deposited in NCBI GenBank under the accession numbers: ON873626–ON873628.
